# Directed functional connectivity of the sensorimotor system in young and older individuals

**DOI:** 10.3389/fnagi.2023.1222352

**Published:** 2023-10-11

**Authors:** Gadi Goelman, Rotem Dan, Ondrej Bezdicek, Robert Jech

**Affiliations:** ^1^Department of Neurology, Ginges Center of Neurogenetics Hadassah Medical Center, Jerusalem, Israel; ^2^Faculty of Medicine, The Hebrew University of Jerusalem, Jerusalem, Israel; ^3^Edmond and Lily Safra Center for Brain Sciences, The Hebrew University of Jerusalem, Jerusalem, Israel; ^4^Department of Neurology and Center of Clinical Neuroscience, Charles University, Prague, Czechia

**Keywords:** aging, functional connectivity, multivariate analysis, neuropsychological tests, dedifferentiation, the impaired GABA theory

## Abstract

**Introduction:**

Studies in the sensorimotor system of older versus young individuals have shown alterations in functional connectivity and organization. Our objective was to explore the implications of these differences in terms of local organizations, and to identify processes that correlate with neuropsychological parameters.

**Methods:**

Using a novel multivariate analysis method on resting-state functional MRI data obtained from 50 young and 31 older healthy individuals, we identified directed 4-node functional pathways within the sensorimotor system and examined their correlations with neuropsychological assessments.

**Results:**

In young individuals, the functional pathways were unidirectional, flowing from the primary motor and sensory cortices to higher motor and visual regions. In older individuals, the functional pathways were more complex. They originated either from the calcarine sulcus or the insula and passed through mutually coupled high-order motor areas before reaching the primary sensory and motor cortices. Additionally, the pathways in older individuals that resembled those found in young individuals exhibited a positive correlation with years of education.

**Discussion:**

The flow pattern of young individuals suggests efficient and fast information transfer. In contrast, the mutual coupling of high-order motor regions in older individuals suggests an inefficient and slow transfer, a less segregated and a more integrated organization. The differences in the number of sensorimotor pathways and of their directionality suggests reduced efferent degenerated pathways and increased afferent compensated pathways. Furthermore, the positive effect of years of education may be associated with the Cognitive Reserve Hypothesis, implying that cognitive reserve could be maintained through specific information transfer pathways.

## 1. Introduction

The sensorimotor system refers to the complex network of structures and processes involved in sensing, perceiving, and controlling movements. With advancing age, the sensorimotor system undergoes various changes, resulting in difficulties in sensory perception, motor control, balance, posture, motor learning, and sensorimotor integration (Varangis et al., 2019; Frolov et al., 2020). The aging process is influenced by a combination of genetic, environmental, and lifestyle factors, and several strategies have been studied to slow down the aging process, such as regular exercise (Rebelo-Marques et al., 2018), healthy diet, adequate sleep (Taillard et al., 2021), stress management (Yegorov et al., 2020), social connections (Van Orden et al., 2021) and mental stimulation (Christie et al., 2017). Multiple macro-scale studies have demonstrated changes in connectivity with age, which can affect the functional communication between different brain regions and contribute to age-related cognitive changes. These changes encompass alterations in structural connectivity, functional connectivity, compensation mechanisms, and disrupted interhemispheric connectivity (Damoiseaux, 2017).

In this study, our aim is to qualitatively compare the macroscopic directed functional connectivity of the sensorimotor system between young and older individuals. Specifically, our objectives are:

(i) To explore the implications of lower segregation, increased integration, and functional dedifferentiation in terms of the differences in local organizations observed in the sensorimotor system of older vs. young individuals (Goh, 2011; Deery et al., 2023). (ii) To identify processes in older individuals that correlate with neuropsychological parameters and to infer their effects on aging. This endeavor aims to unveil the neuronal underpinnings of neuropsychological behaviors through the exploration of directed processes, thereby enriching our comprehension of age-related phenomena. It is worth noting that corresponding scores for the younger individuals are regrettably unavailable. Furthermore, we aim to test and extend our understanding of the impaired GABA (gamma-aminobutyric acid) theory, which suggests that age-related changes in the brain’s primary inhibitory neurotransmitter system, the GABAergic system, contribute to the aging process and age-related cognitive decline (Stagg et al., 2014; Bachtiar et al., 2015).

To obtain precise estimates of directed connectivity, we recently introduced a multivariate analysis method that utilizes resting-state fMRI data. This approach enables the identification of interactions among four different anatomical regions and their directionality using phase coherence, allowing for the definition of functional directed couplings and pathways (Goelman and Dan, 2017; Goelman et al., 2018, 2019, 2021a,b). Computer simulations of the Kuramoto model have been used to test the accuracy of this analysis (Goelman and Dan, 2017), and its applications to the human brain have been demonstrated.

Using resting-state functional MRI data from 50 healthy young and 31 healthy older individuals, we identified functional directed pathways of the sensorimotor system for each group separately. By combining these pathways, we were able to infer general directed flow diagrams for each group. Furthermore, we calculated and combined pathways that correlated with a battery of neuropsychological tests.

## 2. Materials and methods

### 2.1. Subjects

This study used data from young and old healthy participants who underwent fMRI measurements at two different sites using the same systems and identical protocols. The study was approved by the Hadassah Medical Center, Jerusalem, Israel, Ethics Committee and the Ethical Committee of the General University Hospital in Prague, Czech Republic. All participants provided written informed consent prior to inclusion in the study, which was carried out in compliance with the Declaration of Helsinki.

The young subject group: Fifty-two, healthy, young, undergraduate students at the Hebrew University of Jerusalem, Israel were recruited for this study. To exclude past or present psychiatric disorders, participants were evaluated by a clinical psychologist or a psychiatrist using the Structured Clinical Interview for DSM-IV (SCID-5-CV). Additional exclusion criteria were neurological disorders, and, for women, the use of hormonal contraceptives, pregnancy or breastfeeding. Two male subjects were excluded by these criteria, yielding a final sample of 20 men (age:23.9 ± 2.9 years) and 30 women (23.9 ± 2.4 years).

The old subject group: Forty elderly individuals were recruited from a community in Prague. Nine of them were excluded due to: severe atrophy or vascular lesions (*n* = 5), in-scanner motion (*n* = 3), or use of lithium (*n* = 1) which yielded a final sample of 31 individuals. The final group included 16 females (age 61.2 ± 6.4 years) and 15 males (65.2 ± 8.8 years). Exclusion criteria were a history of psychotic symptoms, depression, dementia or a cognitive state on the Montreal Cognitive Assessment <22 (Kopecek et al., 2017).

A segment of this dataset has been previously employed in distinct research endeavors, albeit with varying focuses. Notably, data from the younger subjects were utilized to explore emotional states (Dan et al., 2019a, 2023) and the Default Mode Network (DMN; Goelman et al., 2022), whereas data from the older subjects contributed to investigations related to both the DMN (Goelman et al., 2022) and Parkinson’s diseases (Dan et al., 2019b; Goelman et al., 2021a,b). All participants, in both groups, were right-handed according to the Edinburgh Handedness Inventory.

### 2.2. Neuropsychological tests of older subjects

Older individuals underwent a battery of neuropsychological assessments by an experienced neuropsychologist during a preliminary visit approximately 2 weeks before the MRI session. Depression symptoms were measured using the Beck Depression Inventory (BDI-II; Beck et al., 1996; Ciharova et al., 2020). Anxiety was measured using the Spielberger State–Trait Anxiety Inventory (STAI; Spielberger et al., 1970). Apathy was assessed using the Starkstein Apathy Scale (AS; Starkstein et al., 1992). MCI was estimated using MoCA (Nasreddine et al., 2005; Kopecek et al., 2017) and by a neuropsychological battery to measure cognitive functions (Litvan et al., 2012; Bezdicek et al., 2017; level II, comprehensive assessment, see Supplementary Table 1). Long-term memory was measured by Rey Auditory Verbal Learning Test, delayed recall (Bezdicek et al., 2014) and Brief Visuospatial Memory Test, revised, delayed recall (Benedict et al., 1996; Havlík et al., 2020). The visuospatial function was measured by the CLOX (Royall et al., 1998) and Judgment of Line Orientation (Woodard et al., 1998). Psychomotor speed and working memory were measured by the Trail Making Test, part A (Bezdicek et al., 2012) and by the Digit span backwards from the Wechsler Adult Intelligence Scale, third revision (WAIS-III; Wechsler, 1997). The executive function was measured by the Tower of London (Michalec et al., 2014) and by semantic verbal fluency (Nikolai et al., 2015). Language was measured by the Boston Naming Test, Czech version (Zemanová et al., 2016; Bezdicek et al., 2021) and WAIS-III Similarities (Wechsler, 1997). The score on each test was transformed into a z-score using the Rankit formula (Solomon and Sawilowsky, 2009). Rankits denote the predicted values of order statistics derived from a sample extracted from the standard normal distribution, where the sample size aligns with that of the provided dataset. Among diverse sample sizes and distributions, Rankits have emerged as a notably accurate method. Consequently, they are considered the default choice for score normalization within the realm of social and behavioral sciences (Solomon and Sawilowsky, 2009). The z-scores of each domain were summarized for each individual to create summary scores, with a higher score indicating a better function.

Table 1 lists the participants’ demographic data and the neuropsychological evaluation tests. Supplementary Tables 3, 4 provides further details on the neuropsychological Scores.

**Table 1:** Demographic and neuropsychological characteristics.

Demographics and neuropsychology	Young mean ± SD (range; *n* = 50)	Older mean (raw score) ± SD (range; *n* = 31)
Age, years	23.9 ± 2.6 (19–28)	63.2 ± 7.89 (46–83)
Gender (male/female)	20/30	15/16
Education, years	14.1 ± 1.75 (12–20)	14.8 ± 3.5 (11–25)
BDI-II		6.9 ± 5.2 (0–19)
STAI-X1 (state)		32.7 ± 5.7 (20–47)
STAI-X2 (trait)		35.1 ± 8.2 (22–55)
Starkstein apathy scale		9.58 ± 4.4 (1–21)
MoCA		26.6 ± 2.2 (22–30)
Long-term memory		
RAVLT-30		8.97 **±** 2.5 (2–12)
BVMT-R		10.23 **±** 1.70 (5–12)
Visuospatial function		
Royall’s CLOX (CLOX I)		13.26 ± 1.15 (11–15)
Judgment of line orientation		24.65 ± 3.88 (11–29)
Psychomotor speed and working memory		
Trail making test, part A		35.2 ± 9.1 (20–58)
WAIS-III digit span backwards		6.7 ± 2.2 (4–12)
Executive function		
Tower of London		26.1 ± 4.1 (16–34)
Semantic fluency		65.6 ± 11.8 (38–93)
Language		
WAIS-III similarities		23.7 ± 5.7(11–32)
Boston naming test		54.4 ± 6.5 (27–60)

Abbreviations: BDI-II, Beck Depression Inventory, second edition; BVMT-R, Brief Visuospatial Learning Test—Revised; CLOX, Royall’s Clock Drawing Test; STAI, Spielberger State–Trait Anxiety Inventory; MoCA, Montreal Cognitive Assessment; RAVLT, Rey Auditory Verbal Learning Test; WAIS-III, Wechsler Adult Intelligence Scale—Third Revision.

### 2.3. MRI data acquisition and preprocessing

MRI data of the young and old participants were acquired with 3 T MR scanners (Magnetom Skyra, Siemens, Germany) in the neuroimaging center of the Hebrew University of Jerusalem, Israel; and the Charles University in Prague, Czech Republic, respectively. At both locations, participants underwent a 10-min resting-state fMRI (rs-fMRI) during fixation on a visual crosshair, using the same MRI acquisition protocols. Functional images were acquired using a T2*-weighted gradient-echo, echo-planar imaging sequence with TR = 2 s, TE = 30 ms, image matrix = 64 × 64, field of view = 192 × 192 mm, flip angle = 90°, resolution = 3 × 3 × 3 mm, interslice gap = 0.45 mm. Each brain volume comprised 30 axial slices, and each functional run contained 300 image volumes. Anatomical images were acquired using a sagittal T1-weighted MP-RAGE sequence with TR = 2.2 s, TE = 2.43 ms, resolution = 1 × 1 × 1 mm. For the older participants’ group, T2-weighted images were collected as well, for diagnostic purposes, to exclude significant atrophy or any other pathological brain changes.

All functional MRI data underwent the following preprocessing using SPM12.^1^ Functional images were spatially realigned, coregistered to T1 anatomical images; slice-time corrected and normalized to MNI space. Further preprocessing was done in a CONN toolbox (Whitfield-Gabrieli and Nieto-Castanon, 2012). Potential confounding effects were regressed out using the aCompCor method for anatomical component-based noise correction (Behzadi et al., 2007). These included: (i) outlier scans, i.e., censoring/scrubbing (Power et al., 2014). Outlier scans were identified based on the amount of individual’s motion in the scanner as measured by frame-wise displacement (FD) and global BOLD signals. Acquisitions with FD > 0.9 mm or global BOLD signal changes >5 standard deviations were considered outliers and removed by regression; (ii) the first five principal components (PCAs) of the CSF and white matter signals, to minimize the effects of physiological non-neuronal signals such as cardiac and respiratory signals; (iii) estimated individual’s motion parameters and their first-order derivatives (a total of 12 parameters); (iv) session effects: the potential effects of the beginning of the session were removed by a step function convolved with the hemodynamic response function, in addition to the linear BOLD signal trend. After regression of all potential confounding effects, temporal band-pass filtering (0.008–0.09 Hz) was performed. Note that we did not apply global signal regression, due to its controversy. Global signal regression may introduce artifactual biases (Murphy et al., 2009) and remove potentially meaningful neural components (Chai et al., 2012). Instead, the aCompCor method (Behzadi et al., 2007) was applied to regress out the first 5 principal components (PCAs) of the CSF and white matter signals. This was done to minimize the effects of potential physiological non-neuronal signals such as cardiac and respiratory signals, without the risk of artificially introducing anticorrelations into the functional connectivity estimates.

### 2.4. Multivariate directed functional connectivity and correlations with behavioral tests

A detailed description of the analysis was presented in our previous publications (Goelman and Dan, 2017; Goelman et al., 2018, 2019, 2021a,b), therefore, only the main points are summarized below. For a group of four weakly coupled BOLD temporal signals, with each signal corresponding to a different anatomical location, the analysis assumed that the phases contained temporal information of their mutual coupling. This coupling is expressed in terms of specific relations between the four phases, as we defined (Goelman and Dan, 2017), and enables the definition of four-node pathways corresponding to information transfer among them. Here, we averaged over time and frequency, in the time-frequency wavelet space. Averaging over frequency was performed since in a preliminary study, no effect of frequency was found in the sensorimotor system. Note that we restricted the analysis to continuous, unidirectional pathways to define pathways among the four BOLD signals, i.e., pathways that started in one region and subsequently went through all the other regions. In this case, there were 24 possible pathways (listed in Supplementary Table 5). In this table, the four regions are symbolized by R1 to R4. By choosing pathways that were invariant to the choice of reference-phase (see below), we guaranteed that all phase differences were below 2π (Goelman et al., 2021a,b), thus obtaining unbiased pathways.

For each participant (*sub*) and each pathway’s type (*k*), a binary pathway value (*PW*) was defined as “1” for the cases where phase differences were in line with the pathway and “0” for when they were not (for detailed explanation and illustration, see (Goelman, et al., 2021a; Supplementary Figure 1 their):


(1) $$PW^{k}\left(sub\right)=\left\{\begin{array}{c} 1\\ 0 \end{array}\right\}\begin{array}{c} phasesinlinewiththe\;k\;pathway\;for\;all\;\;\\ \begin{array}{l} 4referencephases\\ phasesnotinlinewiththepathway \end{array} \end{array}$$

with ‘k’ = 1, 2… 24 corresponding to a pathway’s number in Supplementary Table 5, and “*sub*” a subject.

A group pathway index (PWI) was defined as:


(2) $$PWI^{k}=\frac{1}{N}\overset{N}{\underset{i=sub}{\sum }}PW^{k}\left(sub\right)$$

similar to the definition of the phase lag index (PLI; Stam et al., 2007; Stam and van Straaten, 2012) but describing the coherence among four regions, while PLI describes the coherence between two regions. We further note that averaging the wavelet coherences among participants solved the intrinsic time-frequency uncertainty (Torrence and Compo, 1998; Torrence and Webster, 1999).

To identify the pathways in the old subject group that correlated with the nine neuropsychological tests listed in Table 1, we used a partial correlation model between $PW^{k}\left(sub\right)\;$ of Equation 1 and each test, while statistically controlling for the contributions of all other tests, as well as for age, gender, years of education and the frame-wise displacement (FD). This enabled us to obtain the pathways that were uniquely affected by each of these tests. Furthermore, to estimate how essential were specific characteristics of the pathways such as their starting regions (see results), we counted the numbers of the pathways with these characteristics for each individual, and calculated the partial correlations of these sums while controlling for the effects of the other tests and parameters.

Multivariate wavelet calculations were performed with IDL version 8.2.0 (Exelis Visual Information Solutions, Inc.) using custom-developed software. The complex Morlet wavelet functions were chosen for wavelet analysis because they have been shown to provide a good trade-off between time and frequency localization (Muller et al., 2004). We used 10 for the smallest scale, 2 for time resolution, and 21 scales to cover the entire frequency window. Wavelet software was provided by Torrence and Compo (1998) (Torrence and Webster, 1999).^2^

### 2.5. Method workflow

The method workflow consisted of the following steps:

1. Region selection was performed using the Automated Anatomical Labeling (AAL) atlas (Tzourio-Mazoyer et al., 2002), which provided 90 cortical regions. The average BOLD signals were calculated for each individual from each region.
2. To assess the occurrence of these 90 ROIs in sensorimotor pathways, *t*wo preselected sensorimotor regions were chosen, and all possible four-node pathways were calculated by combining these regions with the third and fourth nodes taken from the other 88 AAL ROIs. This resulted in a total of 7,832 pathways. The preselected nodes were the primary sensory and primary motor cortexes of the dominant hemisphere (left).
3. The number of times each of these 88 ROIs appeared within significant pathways (ROI’s rate) was counted separately for the young and older subject groups. Nine regions that were common to both groups and had a high rate were selected.
4. Four-node pathways were calculated for each group separately, considering all possible combinations (330 in total, $11C_{4}$ ) of the selected nine ROIs and the two preselected nodes.
5. The pathways identified in step 4 for the older individuals were examined for significant correlations with the neuropsychological tests.
6. To determine whether pathways in older individuals were similar to those in young individuals, the number of “similar” pathways was partially correlated with each parameter listed in Table 1 (see result “Education affects pathway’ characteristic”).

### 2.6. Statistical analysis

We employed permutation non-parametric tests to calculate the null distributions of Equation 2. The null distributions were obtained using rs-fMRI signals from specific regions of interest (ROIs) selected based on a preliminary study that indicated their high occurrence in sensorimotor pathways. The chosen ROIs were the primary motor, primary sensory, supplementary motor area of the left hemisphere, and the Rolandic operculum of the right hemisphere, as defined by the AAL atlas.

To ensure uncoupling, we utilized a random number generator to select regions from different participants. This process was repeated 10,000 times, and Equation 2 was computed for each group separately. The lowest value of p attainable with 10,000 calculations is 0.0001, thus establishing our cutoff at uncorrected *p* < 0.0001. In the young group, this value corresponded to $PWI^{k}=0.15$ , while in the older subject group, it was $PWI^{k}=0.17.$

It is important to note that in method workflow step 2, where the four-node pathways were calculated 7,832 times, the cutoffs were not corrected for multiple comparisons. However, this step served solely as a guide for ROI selection. In method workflow step numbers four and five, where pathways were calculated 330 times, we applied the Bonferroni correction for multiple comparisons, resulting in cutoffs below $p_{cor}<0.05$ . It is worth mentioning that we used the same uncorrected value of *p* of $10^{-4}\;$ for the partial correlation in step five. As for the correlation in step six, the correlation was performed only once, eliminating the need for correction. For this step, significant results were considered at *p* < 0.05.

### 2.7. Pathway’s directionality

To determine the direction of coupling in the ranked pathways presented in Supplementary Table 5, we made assumptions regarding the signal transfer direction, specifically whether the signal flowed from right to left or from left to right. This is a common consideration in coherent studies and depends on a reference phase, which is an intrinsic factor of the system. Since our pathways were defined for a group, it was necessary for the reference phase to be consistent across all datasets within the group. Therefore, all datasets in a group needed to be acquired using the same system. Once this factor was determined, it was applied to all pathways within the group since it was common to all participants (Stam and van Straaten, 2012).

To infer directionality, we needed to identify a pathway whose directionality was known or could be assumed. Following the methodology of our previous studies (Goelman and Dan, 2017; Goelman et al., 2018, 2019, 2021a,b), we calculated thalamocortical pathways using Equation 2, assuming that the majority of pathways in the resting-state were bottom-up. For these calculations, the preselected regions were the left thalamus, left primary motor cortex, and left primary sensory cortex, focusing on the motor system of the dominant hemisphere. The fourth region consisted of rs-fMRI voxels, utilizing all 3D image voxels. The null distribution of these cases indicated that $PWI^{k}=0.2$ corresponded to cluster-size corrected with *p* ~ 0.001 (Goelman et al., 2021b). Our findings revealed that the majority of the undirected pathways were either “Thalamus-M1-S1-X” or “Thalamus-S1-M1-X,” with “X” representing clusters in the motor system or frontal areas (Goelman et al., 2021a,b). Based on bottom-up processes, we inferred left-to-right directionality in the pathways presented in Supplementary Table 5 for the older group and right-to-left directionality in the pathways for the young group. These directionality assumptions were applied consistently to all pathways in this study.

## 3. Results

### 3.1. ROI’s selection

**Figure 1:**
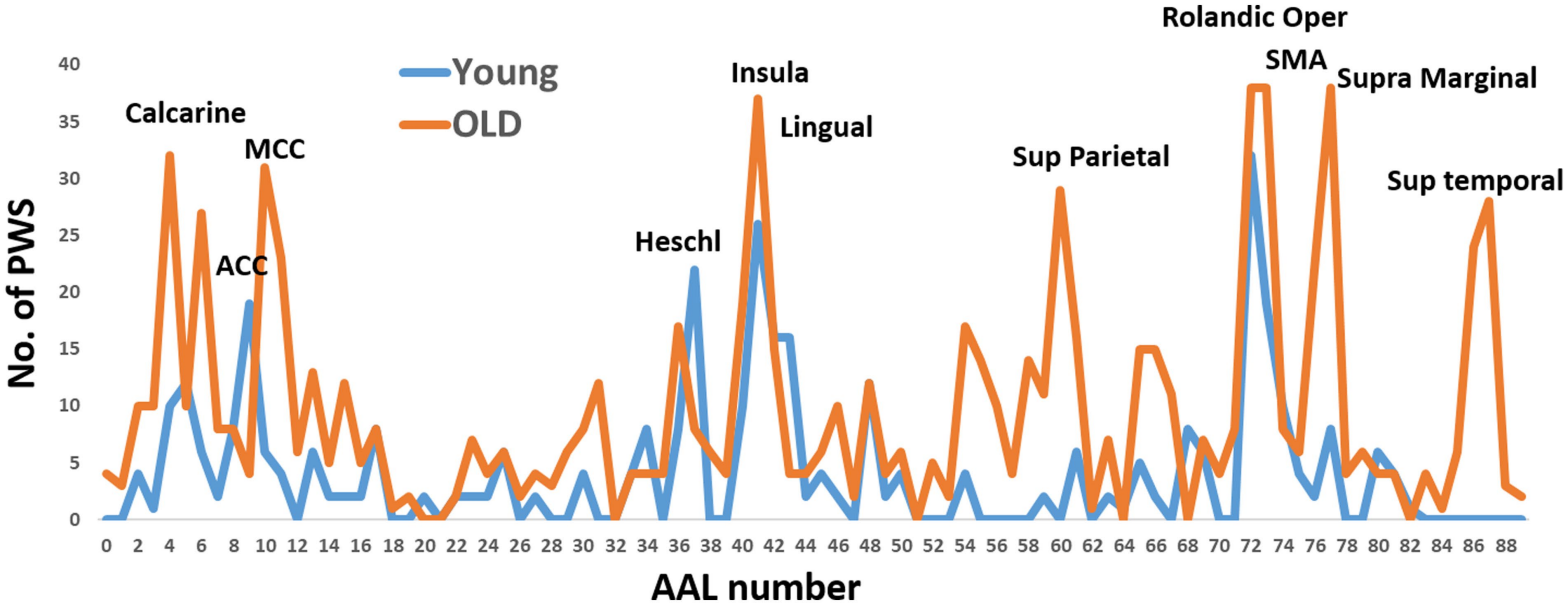
The occurrence of Automated Anatomical Labeling (AAL) regions in directed functional four-node pathways of the young and older subject groups. These pathways were calculated using two predefined nodes: the primary motor and primary sensory cortexes. The x-axis of the graph represents the AAL region numbers.

Using the non-parametric (uncorrected) cutoffs for permutation analysis, we conducted calculations to determine sensorimotor pathways. These pathways consisted of the primary sensory and motor regions in the left hemisphere, along with any of the other 88 AAL ROIs for the third and fourth nodes of the pathways. Our analysis resulted in 434 pathways for older individuals and 185 pathways for young individuals. Figure 1 illustrates the occurrences of ROIs in these pathways, revealing significant differences between the two age groups in the regions involved.

For the young individuals, the regions with the highest occurrence rates were the right anterior cingulate cortex, right Heschel gyrus, right insula, and bilateral Rolandic operculum. On the other hand, the regions with the highest occurrence rates for older individuals were the left calcarine sulcus, left middle cingulate cortex, right insula, bilateral Rolandic operculum, and right supramarginal gyrus. The Rolandic operculum and insula were common regions observed in both age groups.

To simplify the calculations, facilitate interpretations, enable group comparisons, and reduce the number of comparisons, we selected nine ROIs with high or moderate occurrence rates in both groups. These ROIs were combined with the primary motor and sensory regions for the calculations of the four-node pathways in both groups. In other words, we calculated all possible permutations of these 11 ROIs, resulting in 330 pathways. The following ROIs were chosen: left calcarine sulcus, left middle cingulate cortex (MCC), bilateral insula, right lingual gyrus, bilateral Rolandic operculum, left supplementary motor area (SMA), and right supramarginal gyrus.

### 3.2. The young subjects’ group

**Figure 2:**
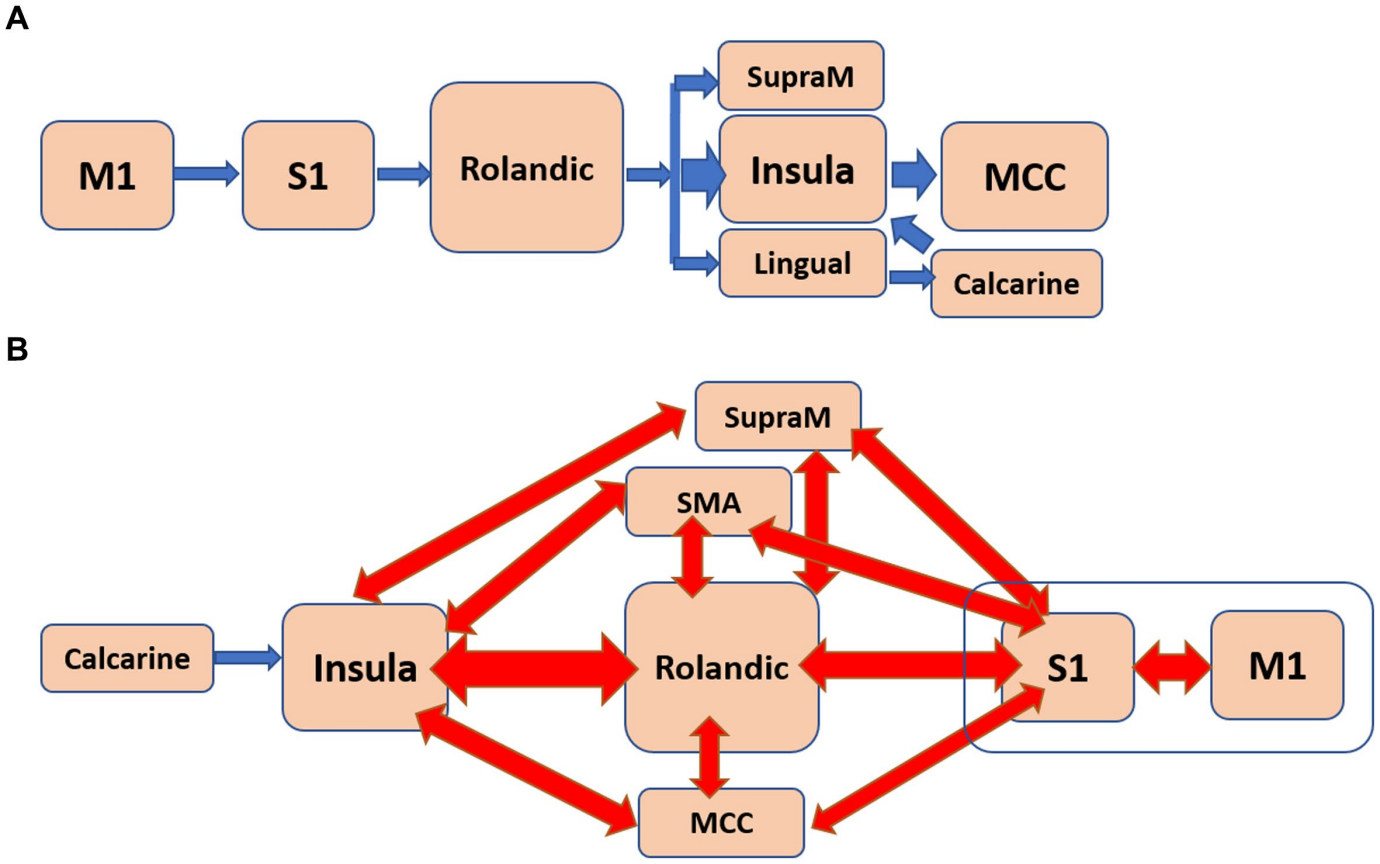
The estimated flow group diagrams illustrate the consolidation of most of the pathways in each group. **(A)**. Flow diagram of the young individuals. **(B)**. Flow diagram of the older individuals. Arrows in blue indicate unidirectional connections, while arrows in red represent bidirectional connections.

Table 2 presents a list of the 42 pathways that exhibited significance in young individuals. Among these pathways, 28 initiated from the primary motor cortex, while 10 originated from the primary sensory cortex. Notably, 20 pathways terminated in the insula. To create an estimated flow diagram consolidating these pathways, certain considerations were made. Firstly, it was acknowledged that these pathways represented functional connections rather than precise anatomical connections and second, that they were assumed to be strictly unidirectional. Consequently, we applied linear arguments to merge pathways together. For instance, the pathways “A → B → C → D” and “B → C → D → E” were merged into the pathway “A → B → C → D → E”. Similarly, the pathways “A → B → C → D” and “A → B → D → C” were combined into “A → B → [C↔D]”, indicating bidirectional communication. By employing such inferences, we derived a generalized resting-state sensorimotor flow for young individuals from the information in Table 2, as depicted in Figure 2A. To elucidate, these assumptions are linear in nature and were applied to the directed four-node pathways, potentially resulting in partial accuracy. This flow pattern suggested a feedforward progression that initiated from the primary motor and sensory cortices, traversed the Rolandic operculum and insula, and reached either the cingulate or calcarine sulcus. This direct and feedforward flow is anticipated to be rapid in nature.

**Table 2:** List of pathways identified in the young subject group.

Region 1	Region 2	Region 3	Region 4
Precentral_L	Rolandic_Oper_L	Rolandic_Oper_R	Insula_L
Precentral_L	Rolandic_Oper_L	Rolandic_Oper_R	Insula_R
Precentral_L	Rolandic_Oper_L	Supp_Motor_Area_L	Insula_R
Precentral_L	Postcentral_L	Insula_R	Insula_L
Precentral_L	Postcentral_L	Rolandic_Oper_L	Insula_L
Precentral_L	Postcentral_L	Rolandic_Oper_L	Insula_R
Precentral_L	Postcentral_L	Rolandic_Oper_R	Insula_R
Precentral_L	Rolandic_Oper_L	Insula_L	Insula_R
Precentral_L	Rolandic_Oper_R	Insula_L	Insula_R
Precentral_L	Supp_Motor_Area_L	Insula_L	Insula_R
Precentral_L	Rolandic_Oper_R	Insula_R	Insula_L
Precentral_L	Rolandic_Oper_R	Rolandic_Oper_L	Insula_L
Precentral_L	Rolandic_Oper_R	Rolandic_Oper_L	Insula_R
Precentral_L	Supp_Motor_Area_L	Rolandic_Oper_R	Insula_R
Precentral_L	Rolandic_Oper_L	Insula_L	Precentral_L
Precentral_L	Rolandic_Oper_R	Insula_L	Cingulum_Mid_L
Precentral_L	Rolandic_Oper_L	Insula_R	Cingulum_Mid_L
Precentral_L	Rolandic_Oper_R	Insula_R	Cingulum_Mid_L
Precentral_L	Insula_L	Insula_R	Cingulum_Mid_L
Precentral_L	Postcentral_L	Rolandic_Oper_L	SupraMarginal_R
Precentral_L	Postcentral_L	Rolandic_Oper_R	SupraMarginal_R
Precentral_L	Rolandic_Oper_L	Rolandic_Oper_R	SupraMarginal_R
Precentral_L	Rolandic_Oper_L	Lingual_R	Calcarine_L
Precentral_L	Rolandic_Oper_R	Lingual_R	Calcarine_L
Precentral_L	Supp_Motor_Area_L	Lingual_R	Calcarine_L
Precentral_L	Postcentral_L	Insula_R	Supp_Motor_Area_L
Precentral_L	Postcentral_L	Rolandic_Oper_L	Rolandic_Oper_R
Precentral_L	Postcentral_L	Rolandic_Oper_L	Lingual_R
Postcentral_L	Rolandic_Oper_L	Insula_R	Cingulum_Mid_L
Postcentral_L	Rolandic_Oper_R	Insula_R	Cingulum_Mid_L
Postcentral_L	Rolandic_Oper_R	Rolandic_Oper_L	Cingulum_Mid_L
Postcentral_L	Rolandic_Oper_L	Rolandic_Oper_R	SupraMarginal_R
Postcentral_L	Rolandic_Oper_L	Lingual_R	Calcarine_L
Postcentral_L	Rolandic_Oper_L	Rolandic_Oper_R	Insula_L
Postcentral_L	Rolandic_Oper_L	Rolandic_Oper_R	Insula_R
Postcentral_L	Rolandic_Oper_L	Insula_L	Insula_R
Postcentral_L	Supp_Motor_Area_L	Insula_L	Insula_R
Postcentral_L	Supp_Motor_Area_L	Rolandic_Oper_R	Insula_R
Lingual_R	Calcarine_L	Rolandic_Oper_R	Cingulum_Mid_L
Lingual_R	Calcarine_L	Insula_L	Cingulum_Mid_L
Lingual_R	Calcarine_L	Insula_R	Cingulum_Mid_L
Lingual_R	Calcarine_L	Insula_R	Insula_L

The directionality is from left to right, starting from region 1, then progressing to region 2, region 3, and finally reaching region 4.

### 3.3. The older subjects’ group

Table 3 lists the 135 pathways that demonstrated significance in older individuals. Among these pathways, 57 originated from the insula, 30 from the calcarine sulcus, 19 from the middle cingulate cortex, and 17 from the supramarginal gyrus. Notably, 55 pathways terminated in the primary motor area, while 51 pathways ended in the primary sensory area. The complexity of the flow depicted in Table 3 made it challenging to infer a single flow diagram that encompassed all pathways.

Figure 2B represents a suggested flow diagram that aligns with the majority of pathways presented in Table 3. It reveals a predominantly bidirectional network involving the Rolandic operculum, supplementary motor area (SMA), supramarginal gyrus, middle cingulate cortex (MCC), and insula. This organization suggests a less specific arrangement, often referred to as “dedifferentiation,” with information flow likely occurring at a slower pace. Furthermore, the directionality of the flow generally opposes that observed in young individuals. In this case, the flow terminates in the sensorimotor regions while commencing from various locations throughout the brain.

**Table 3:** List of pathways identified in the older subject group.

Region 1	Region 2	Region 3	Region 4
Insula_L	Insula_R	Rolandic_Oper_R	Precentral_L
Insula_L	Rolandic_Oper_L	Rolandic_Oper_R	Precentral_L
Insula_L	Rolandic_Oper_L	Supp_Motor_Area_L	Precentral_L
Insula_L	Insula_R	Rolandic_Oper_L	Postcentral_L
Insula_L	Insula_R	Rolandic_Oper_R	Postcentral_L
Insula_L	Rolandic_Oper_L	Rolandic_Oper_R	Postcentral_L
Insula_L	Rolandic_Oper_L	Precentral_L	Postcentral_L
Insula_L	SupraMarginal_R	Rolandic_Oper_R	Precentral_L
Insula_L	SupraMarginal_R	Rolandic_Oper_L	Postcentral_L
Insula_L	Supp_Motor_Area_L	Rolandic_Oper_R	Postcentral_L
Insula_L	SupraMarginal_R	Rolandic_Oper_R	Postcentral_L
Insula_L	Rolandic_Oper_L	Postcentral_L	Precentral_L
Insula_L	Rolandic_Oper_R	Postcentral_L	Precentral_L
Insula_L	Supp_Motor_Area_L	Cingulum_Mid_L	Postcentral_L
Insula_L	Cingulum_Mid_L	Postcentral_L	Precentral_L
Insula_L	Postcentral_L	Supp_Motor_Area_L	Precentral_L
Insula_L	Cingulum_Mid_L	Rolandic_Oper_R	Precentral_L
Insula_L	Calcarine_L	Rolandic_Oper_R	Postcentral_L
Insula_L	Cingulum_Mid_L	Rolandic_Oper_L	Postcentral_L
Insula_L	Cingulum_Mid_L	Rolandic_Oper_R	Postcentral_L
Insula_L	Rolandic_Oper_L	Postcentral_L	Supp_Motor_Area_L
Insula_R	Rolandic_Oper_L	Supp_Motor_Area_L	Precentral_L
Insula_R	Rolandic_Oper_R	Supp_Motor_Area_L	Precentral_L
Insula_R	Rolandic_Oper_L	Rolandic_Oper_R	Postcentral_L
Insula_R	Rolandic_Oper_L	Precentral_L	Postcentral_L
Insula_R	Rolandic_Oper_R	Precentral_L	Postcentral_L
Insula_R	Rolandic_Oper_R	Rolandic_Oper_L	Precentral_L
Insula_R	SupraMarginal_R	Rolandic_Oper_R	Precentral_L
Insula_R	Rolandic_Oper_R	Rolandic_Oper_L	Postcentral_L
Insula_R	Supp_Motor_Area_L	Rolandic_Oper_L	Postcentral_L
Insula_R	Insula_L	Precentral_L	Postcentral_L
Insula_R	Rolandic_Oper_L	Postcentral_L	Precentral_L
Insula_R	Rolandic_Oper_R	Postcentral_L	Precentral_L
Insula_R	Rolandic_Oper_L	Cingulum_Mid_L	Precentral_L
Insula_R	SupraMarginal_R	Cingulum_Mid_L	Precentral_L
Insula_R	Rolandic_Oper_R	Insula_L	Precentral_L
Insula_R	Rolandic_Oper_R	Cingulum_Mid_L	Postcentral_L
Insula_R	SupraMarginal_R	Cingulum_Mid_L	Postcentral_L
Insula_R	Cingulum_Mid_L	Postcentral_L	Precentral_L
Insula_R	Insula_L	Postcentral_L	Precentral_L
Insula_R	Cingulum_Mid_L	Calcarine_L	Precentral_L
Insula_R	Insula_L	Cingulum_Mid_L	Precentral_L
Insula_R	Insula_L	Cingulum_Mid_L	Postcentral_L
Insula_R	Calcarine_L	Rolandic_Oper_L	Precentral_L
Insula_R	Cingulum_Mid_L	Rolandic_Oper_R	Precentral_L
Insula_R	Insula_L	Rolandic_Oper_L	Precentral_L
Insula_R	Insula_L	Rolandic_Oper_R	Precentral_L
Insula_R	Insula_L	Supp_Motor_Area_L	Precentral_L
Insula_R	Cingulum_Mid_L	Rolandic_Oper_L	Postcentral_L
Insula_R	Insula_L	Rolandic_Oper_L	Postcentral_L
Insula_R	Insula_L	Rolandic_Oper_R	Postcentral_L
Insula_R	Rolandic_Oper_R	Precentral_L	Supp_Motor_Area_L
Insula_R	Rolandic_Oper_R	Postcentral_L	Supp_Motor_Area_L
Insula_R	Insula_L	Precentral_L	Supp_Motor_Area_L
Insula_R	Insula_L	Postcentral_L	Supp_Motor_Area_L
Insula_R	Insula_L	Lingual_R	Calcarine_L
Insula_R	Precentral_L	Calcarine_L	Lingual_R
Calcarine_L	Cingulum_Mid_L	Insula_L	Rolandic_Oper_L
Calcarine_L	Cingulum_Mid_L	Rolandic_Oper_L	Precentral_L
Calcarine_L	Cingulum_Mid_L	Rolandic_Oper_R	Precentral_L
Calcarine_L	Insula_L	Rolandic_Oper_L	Precentral_L
Calcarine_L	Insula_R	Rolandic_Oper_L	Precentral_L
Calcarine_L	Lingual_R	Rolandic_Oper_L	Precentral_L
Calcarine_L	Lingual_R	Supp_Motor_Area_L	Precentral_L
Calcarine_L	Rolandic_Oper_L	Supp_Motor_Area_L	Precentral_L
Calcarine_L	Cingulum_Mid_L	Insula_L	Postcentral_L
Calcarine_L	Cingulum_Mid_L	Rolandic_Oper_L	Postcentral_L
Calcarine_L	Cingulum_Mid_L	Rolandic_Oper_R	Postcentral_L
Calcarine_L	Cingulum_Mid_L	Supp_Motor_Area_L	Postcentral_L
Calcarine_L	Insula_L	Rolandic_Oper_L	Postcentral_L
Calcarine_L	Insula_R	Rolandic_Oper_L	Postcentral_L
Calcarine_L	Insula_R	Rolandic_Oper_R	Postcentral_L
Calcarine_L	Lingual_R	Supp_Motor_Area_L	Postcentral_L
Calcarine_L	Insula_R	Precentral_L	Postcentral_L
Calcarine_L	Cingulum_Mid_L	Rolandic_Oper_R	Rolandic_Oper_L
Calcarine_L	Cingulum_Mid_L	Supp_Motor_Area_L	Rolandic_Oper_R
Calcarine_L	SupraMarginal_R	Cingulum_Mid_L	Precentral_L
Calcarine_L	Insula_R	Insula_L	Precentral_L
Calcarine_L	Rolandic_Oper_R	Rolandic_Oper_L	Precentral_L
Calcarine_L	Insula_R	Insula_L	Postcentral_L
Calcarine_L	Rolandic_Oper_R	Rolandic_Oper_L	Postcentral_L
Calcarine_L	Supp_Motor_Area_L	Rolandic_Oper_L	Postcentral_L
Calcarine_L	Cingulum_Mid_L	Postcentral_L	Precentral_L
Calcarine_L	Lingual_R	Postcentral_L	Precentral_L
Calcarine_L	Rolandic_Oper_R	Postcentral_L	Precentral_L
Calcarine_L	Lingual_R	Cingulum_Mid_L	Supp_Motor_Area_L
Calcarine_L	SupraMarginal_R	Cingulum_Mid_L	Rolandic_Oper_R
Cingulum_Mid_L	Rolandic_Oper_L	Rolandic_Oper_R	Postcentral_L
Cingulum_Mid_L	Rolandic_Oper_R	Supp_Motor_Area_L	Postcentral_L
Cingulum_Mid_L	Insula_L	Lingual_R	Calcarine_L
Cingulum_Mid_L	Insula_R	Lingual_R	Calcarine_L
Cingulum_Mid_L	Rolandic_Oper_R	Rolandic_Oper_L	Precentral_L
Cingulum_Mid_L	SupraMarginal_R	Lingual_R	Postcentral_L
Cingulum_Mid_L	Rolandic_Oper_R	Rolandic_Oper_L	Postcentral_L
Cingulum_Mid_L	Supp_Motor_Area_L	Rolandic_Oper_L	Postcentral_L
Cingulum_Mid_L	SupraMarginal_R	Rolandic_Oper_L	Postcentral_L
Cingulum_Mid_L	Supp_Motor_Area_L	Rolandic_Oper_R	Postcentral_L
Cingulum_Mid_L	SupraMarginal_R	Rolandic_Oper_R	Postcentral_L
Cingulum_Mid_L	Precentral_L	Postcentral_L	Rolandic_Oper_L
Cingulum_Mid_L	Rolandic_Oper_L	Postcentral_L	Precentral_L
Cingulum_Mid_L	Rolandic_Oper_R	Postcentral_L	Precentral_L
Cingulum_Mid_L	Supp_Motor_Area_L	Postcentral_L	Precentral_L
Cingulum_Mid_L	Rolandic_Oper_L	Precentral_L	Lingual_R
Cingulum_Mid_L	Supp_Motor_Area_L	Postcentral_L	Insula_L
Cingulum_Mid_L	Supp_Motor_Area_L	Postcentral_L	Rolandic_Oper_L
Cingulum_Mid_L	Supp_Motor_Area_L	Postcentral_L	Rolandic_Oper_R
SupraMarginal_R	Insula_R	Precentral_L	Postcentral_L
SupraMarginal_R	Postcentral_L	Precentral_L	Supp_Motor_Area_L
SupraMarginal_R	Insula_R	Precentral_L	Supp_Motor_Area_L
SupraMarginal_R	Rolandic_Oper_R	Precentral_L	Supp_Motor_Area_L
SupraMarginal_R	Insula_R	Postcentral_L	Supp_Motor_Area_L
SupraMarginal_R	Cingulum_Mid_L	Postcentral_L	Precentral_L
SupraMarginal_R	Insula_L	Postcentral_L	Precentral_L
SupraMarginal_R	Insula_R	Postcentral_L	Precentral_L
SupraMarginal_R	Rolandic_Oper_L	Postcentral_L	Precentral_L
SupraMarginal_R	Rolandic_Oper_R	Postcentral_L	Precentral_L
SupraMarginal_R	Insula_L	Cingulum_Mid_L	Precentral_L
SupraMarginal_R	Insula_R	Cingulum_Mid_L	Precentral_L
SupraMarginal_R	Insula_R	Insula_L	Precentral_L
SupraMarginal_R	Rolandic_Oper_R	Rolandic_Oper_L	Precentral_L
SupraMarginal_R	Insula_R	Insula_L	Postcentral_L
SupraMarginal_R	Rolandic_Oper_R	Rolandic_Oper_L	Postcentral_L
SupraMarginal_R	Supp_Motor_Area_L	Rolandic_Oper_L	Postcentral_L
Rolandic_Oper_R	Rolandic_Oper_L	Postcentral_L	Supp_Motor_Area_L
Rolandic_Oper_R	Rolandic_Oper_L	Postcentral_L	Precentral_L
Rolandic_Oper_R	Postcentral_L	Lingual_R	Calcarine_L
Rolandic_Oper_L	Postcentral_L	Precentral_L	Supp_Motor_Area_L
Supp_Motor_Area_L	Rolandic_Oper_R	Rolandic_Oper_L	Postcentral_L
Supp_Motor_Area_L	Postcentral_L	Lingual_R	Cingulum_Mid_L
Lingual_R	Supp_Motor_Area_L	Precentral_L	Postcentral_L
Lingual_R	Insula_R	Rolandic_Oper_L	Postcentral_L
Lingual_R	Insula_R	Rolandic_Oper_R	Postcentral_L
Insula_R	Insula_L	Rolandic_Oper_R	Precentral_L
Precentral_L	Supp_Motor_Area_L	Lingual_R	Calcarine_L

The directionality is from left to right, starting from region 1, then progressing to region 2, region 3, and finally reaching region 4.

### 3.4. Correlation with neuropsychological assessments

**Figure 3:**
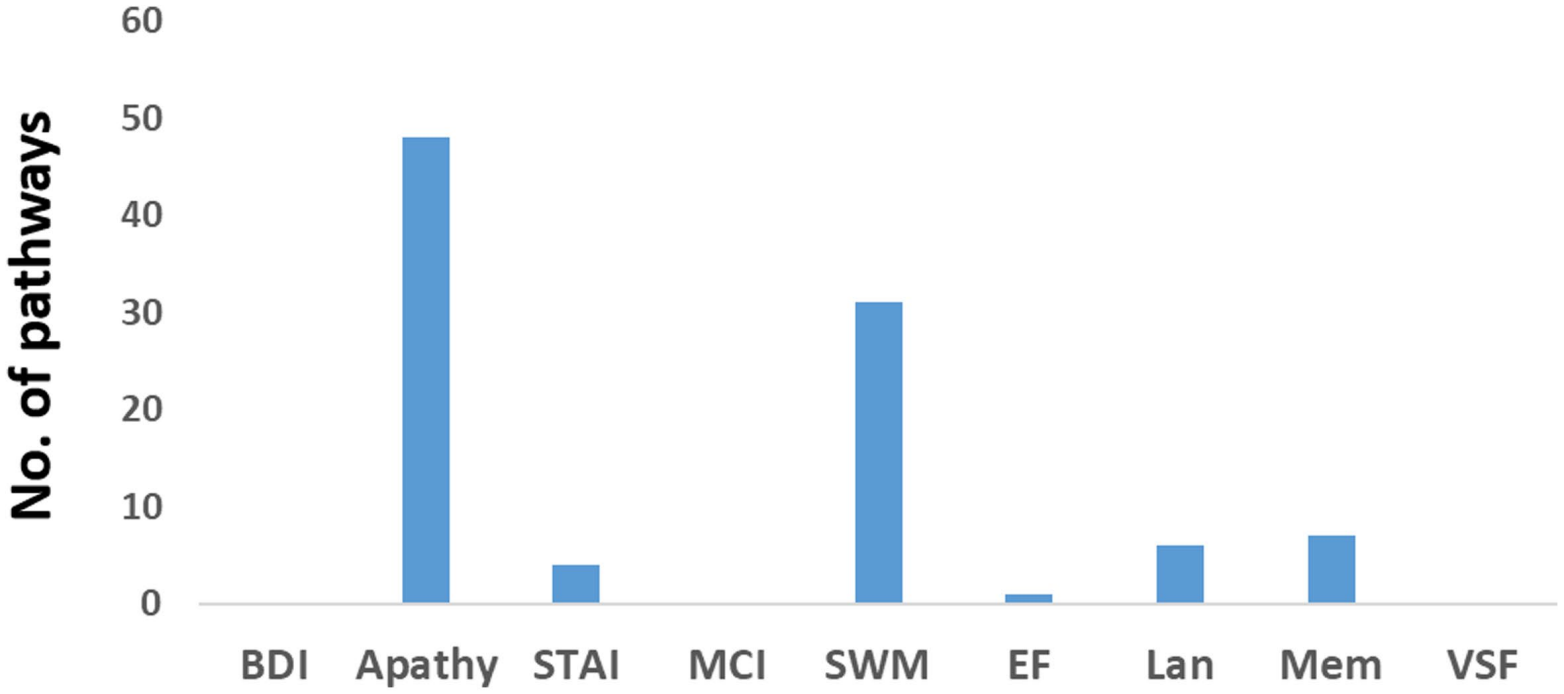
Number of pathways in the older individuals that exhibited a positive correlation with various neuropsychological assessments. BDI refers to the Beck Depression Inventory, STAI represents the State–Trait Anxiety Inventory, MCI indicates mild cognitive impairment, SWM represents psychomotor speed and working memory, EF refers to executive function, Lan represents language, Mem denotes memory, and VSF represents visuospatial function.

Figure 3 displays the number of pathways in the older individuals exhibiting significant positive correlations with neuropsychological assessments. Only a small number of pathways (less than 6) were found to have a negative correlation. Among the positive correlations, the assessments of apathy and psychomotor-speed-and-working-memory (SWM), exhibited a high number of pathways. Detailed information regarding these pathways can be found in Supplementary Tables 6, 7, which highlight their intricate nature.

**Figure 4:**
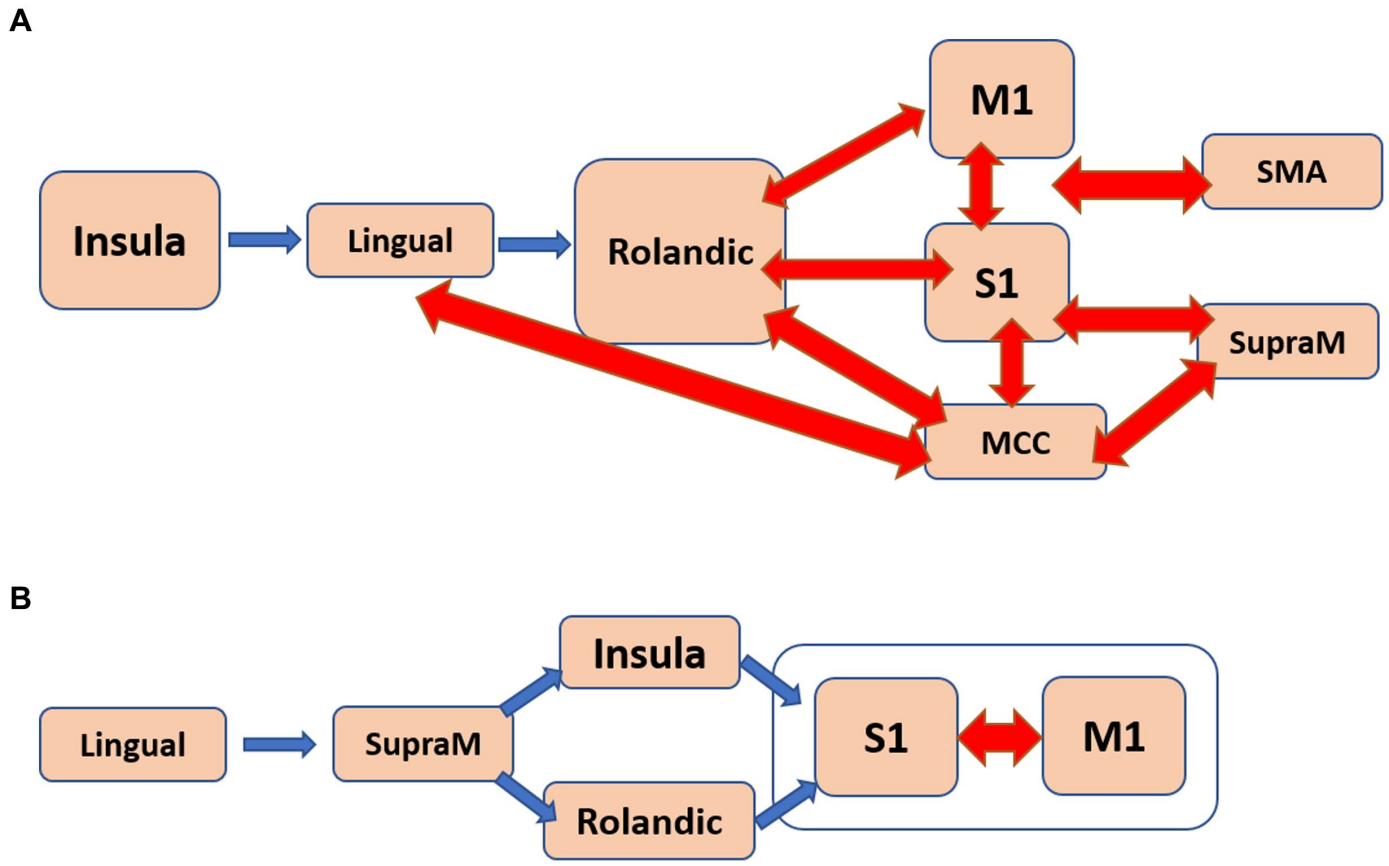
The estimated flow diagrams for the older individuals illustrate the consolidation of most of the pathways that positively correlate with: **(A)**. Apathy. **(B)**. Psychomotor speed and working memory. Arrows in blue indicate unidirectional connections, while arrows in red represent bidirectional connections.

Regarding apathy, a total of 48 pathways were identified. Among them, 18 pathways originated from the insula, eight from the middle cingulate cortex (MCC), and eight from the primary motor cortex (M1). Figure 4A presents a flow diagram that summarizes the majority of pathways associated with apathy. It illustrates a complex information flow pattern that initiates from the insula and involves mutual interactions between all sensorimotor regions.

In the case of SWM, 31 pathways showed correlation. Among them, nine pathways started in the lingual gyrus, six in the Rolandic operculum, and five in the MCC. The pathways originating from the lingual gyrus terminated in either the primary somatosensory cortex (S1) or the primary motor cortex (M1). Notably, the pathways correlated with SWM seemed to correspond to at least two distinct flow diagrams. One of them, depicted in Figure 4B, involved a unidirectional flow from the lingual gyrus through the supramarginal sulcus to the primary sensorimotor cortex. The other diagram featured complex interactions between the MCC, Rolandic operculum, insula, and SMA.

### 3.5. Education affects pathway’ characteristic

The striking disparities observed in Figure 2, highlighting the differences in pathways between young and older individuals, led us to investigate whether there were any pathways in older individuals that resembled those of the younger individuals. If such pathways existed, we aimed to characterize them. To explore this, we examined whether the number of pathways in older individuals starting from either the primary motor or sensory cortexes correlated with any demographic or neuropsychological characteristics listed in Table 1.

To accomplish this, we quantified the number of pathways originating from the primary motor or sensory cortex in each individual and calculated the partial correlation between these counts and each parameter from Table 1. It is important to note that this count was not limited to the significant pathways in Supplementary Table 5 but encompassed all 330 calculated pathways. We conducted statistical corrections to account for the influence of other parameters. Remarkably, only one significant correlation emerged, which was with the variable ‘years of education.’ This correlation exhibited a positive relationship with an R-value of 0.4 (*p* < 0.03).

## 4. Discussion

This study focuses on investigating the sensorimotor functional organization in resting-state conditions among young and older healthy individuals. The objective is to gain a better understanding of the differences and similarities between these age groups. This research builds upon our previous study, which revealed opposing flow directions between the default-mode network (DMN) and the sensorimotor networks. Specifically, in young individuals, pathways combined the sensorimotor (SM) and the DMN in an efferent manner (DMN < SM), while in older individuals, the flow was afferent (DMN > SM; Goelman et al., 2022). In here, we employed a simplified version (averaged over frequency) of our novel method of multivariate directed functional connectivity (Goelman and Dan, 2017; Goelman et al., 2018, 2019, 2021a,b). This method, based on phase coherences, enables the inference of multiple-node directed functional pathways, providing insights into the flow of information between anatomical regions.

Our study’s key findings are as follows:

- The older individuals exhibited a higher number of sensorimotor pathways compared to the young individuals (more than three times), indicating greater functional connectivity in the older age group.
- In young individuals, the functional flow demonstrated a unidirectional pattern, originating from the primary motor and sensory cortices and extending to higher-level motor and visual regions (Figure 2A). This “simple” flow is expected to be efficient and fast.
- In contrast, the functional flow in older individuals was considerably more complex. It originated from the calcarine sulcus or the insula, passed through high-order motor areas characterized by mutually involved bidirectional connections, and ultimately reached the primary sensory and motor cortices (Figure 2B). This organization is anticipated to be less efficient, slower, and less specific.
- The older individuals displayed a significant number of pathways that positively correlated with apathy and with SWM (Figure 3). In the case of apathy, these pathways initiated in the insula and exhibited complex bidirectional flow between primary and higher-order motor and sensory regions (Figure 4A). For SWM, the pathways suggested several distinct processes, with the most frequent one involving unidirectional flow from the lingual gyrus to the primary sensorimotor cortex (Figure 4B).
- Pathways in older individuals that shared similarities with pathways in young individuals in terms of their starting regions, exhibited a positive correlation with “years of education.”

The finding of a higher number of sensorimotor pathways in older individuals, indicating higher functional connectivity, aligns with previous reports. A literature review on large-scale resting-state functional brain networks across the adult lifespan demonstrated that older adults exhibit reduced within-network connectivity but increased between-network connectivity (Deery et al., 2023). Additionally, studies have reported lower levels of endogenous γ-aminobutyric acid (GABA) in the resting sensorimotor system of older individuals, and these GABA levels were found to be negatively correlated with the strength of resting functional motor connectivity (Stagg et al., 2014; Bachtiar et al., 2015). Several studies have investigated alterations in effective connections within motor networks through the application of directed connectivity analysis mainly during motor execution. Notably, Wang and colleagues employed Granger causality and unveiled heightened effective connections among motor regions, coupled with diminished within-hemisphere connections in older individuals (Wang et al., 2019). Likewise, a study utilizing magnetoencephalography data and Granger causality demonstrated an escalation in both functional and effective connectivity throughout the entire brain in older subjects. This entailed an augmentation in contralateral information flow and a concurrent reduction in interhemispheric flow (Larivière et al., 2019).

On the behavioral level, age-related motor slowdown, increased reaction time, reduced motor control, and difficulties in learning new motor skills were reported (Frolov et al., 2020). These findings are consistent with the findings of complex flow organizations found for the older individuals that include complex bidirectional connections. Furthermore, the mutual coupling of high-order motor regions suggests a less segregated and more integrated organization, which aligns with findings of functional dedifferentiation and altered connectivity in older adults (Goh, 2011).

The unique capacity of our method to discern the directionality of flow has empowered us to pinpoint age-dependent pathways, which we theorize might embody degenerative and compensatory processes. Specifically, the diminished count of sensorimotor efferent pathways in the older individual group is posited to reflect a degenerative progression, leading to the reduction or elimination of pathways that were evident in the younger individual group. Similarly, the elevated number of sensorimotor afferent pathways in the older individual group is perceived as indicative of supplementary, compensatory processes that were not prominent in the younger subject group.

This speculation aligns seamlessly with the broader context of sensorimotor efferent processes, encompassing facets such as motor control, coordination, execution of motor commands, motor learning, and the acquisition of novel motor skills, all of which tend to decline with advancing age (Frolov et al., 2020). In contrast, older individuals might counterbalance these deficits by emphasizing skills tied to sensorimotor afferent processes, encompassing sensory perception (e.g., vision and hearing) and sensory processing (e.g., discrimination or integration of sensory inputs). In this context, we suggest that the reduction in sensorimotor efferent activity correspond to degenerated pathways, while the heightened sensorimotor afferent activity to adaptive compensatory mechanisms.

With regards to the correlation with neuropsychological assessments in the older subjects, we showed that while apathy is primarily associated with changes in mood and motivation, it can also affect the sensorimotor system of older individuals, leading to reduced goal-directed behavior and motor slowing or impairments in motor planning. Figure 4A, the estimated flow diagrams that is correlated with apathy, illustrates the mutual influence of multiple high-order sensorimotor regions on the primary motor and sensory cortices, which aligns with the concept of slowness and difficulties in goal-directed behavior. The critical involvement of the insula in apathy has been demonstrated previously, including the directionality depicted in Figure 4A (Goelman et al., 2021b). Furthermore, elderly individuals often exhibit slow response and processing, difficulties in motor coordination, reduced working memory capacity and efficiency, and increased susceptibility to interferences. Supplementary Table 7 illustrates several distinct processes that were correlated with SWM, with the flow depicted in Figure 4B being the most frequently observed. This flow pattern resembles the expected information flow during working memory tasks, involving observations leading to actions.

Finally, the finding of a positive correlation between the number of pathways starting in the primary sensorimotor system in older individuals and “years of education” may be attributed to the Cognitive Reserve Hypothesis (CRH; Tucker-Drob et al., 2009). According to the CRH, individuals with higher cognitive reserve possess a greater ability to resist cognitive decline. Cognitive reserve refers to the brain’s capacity to optimize or adapt its cognitive processes and is primarily focused on the relationship between cognitive reserve and cognitive abilities. However, it is important to recognize that the sensorimotor system, which encompasses sensory perception and motor control, can also contribute to the cognitive reserve framework. Several studies have indicated a connection between the sensorimotor system and cognitive reserve (Guzzetti et al., 2019). Hence, we speculate that cognitive reserve may also manifest in the sensorimotor pathways that resemble those found in young individuals. This suggests that the preservation of specific information-transfer pathways within the sensorimotor system could contribute to cognitive reserve.

How can the confluence of the aforementioned findings be synthesized into a unified framework? One potential avenue is through the lens of the GABA deficit theory (Rozycka and Liguz-Lecznar, 2017), although our study indirectly aligns with this theory due to the absence of GABA level measurements. The GABA deficit theory posits that functional impairments in GABA-mediated neural signaling constitute a mechanism underlying age-related declines in behavior (Rozycka and Liguz-Lecznar, 2017), neural distinctiveness in perceptual and motor realms (Cassady et al., 2020), and alterations in functional dedifferentiation and connectivity patterns (Goh, 2011). Supporting this theory, imbalanced GABAergic concentrations have been implicated in impaired behavior (Heise et al., 2013), increased connectivity, reduced segregation, enhanced functional dedifferentiation, and compromised motor control (Hamer and Chida, 2009; Heise et al., 2022). Notably, many of our findings corroborate the GABA deficit theory, deepening our comprehension of phenomena such as reduced segregation and heightened dedifferentiation. The alteration in sensorimotor directionality observed in our study may also be intricately linked to the GABA deficit theory, albeit in a more intricate manner. This shift in directionality is hypothesized to reflect a blend of degenerative and compensatory processes. Degenerative changes might involve neuronal loss, structural and functional shifts in neural networks, and a decline in neurotransmitter levels, such as GABA. Figure 2B posits that the compensatory strategy in older individuals encompasses reciprocal activation of brain regions and the integration of others that once served distinct functions. This adaptive process culminates in dedifferentiation, a phenomenon associated with compromised GABA concentrations.

An alternative framework for unifying all these findings, particularly those delineated in Figure 2, draws parallels with our previous investigation. In that study, we elucidated divergent directionality patterns between the sensorimotor and Default Mode (DM) networks across young and older cohorts. Specifically, pathways fusing the sensorimotor (SM) and DM networks displayed efferent directionality in the young group (DMN < SM), while afferent directionality was evident in the older group (DMN > SM). We theorized that this directional contrast could be attributed to heightened regulatory influence emanating from the medial prefrontal cortex on sensorimotor activity (Goelman et al., 2022). Applying a similar rationale, we postulate that reciprocal interactions within several higher-order motor regions—encompassing components of the cingulate, supplementary motor area (SMA), rolandic operculum, and insula—may function analogously to the medial prefrontal cortex in governing other motor functions that could also be non-voluntary activity. Herein, we do not have experimental findings to support these speculative ideas. To substantiate any of these conceptual views, further empirical investigations are imperative.

By employing a measure of multiple-node directed functional pathways, we were able to identify specific differences between young and older individuals, in their functional organizations that explain the higher integration, dedifferentiation, and inefficient information transfer in the sensorimotor system of older individuals. Additionally, we identified specific pathways that are reserved by cognitive processes. This approach enhances our understanding of the underlying age-dependent mechanisms in the sensorimotor system.

### 4.1. Limitation

We recognize that the varying sample sizes within our study may introduce bias. To mitigate these potential biases, we incorporated sample size considerations into our null distribution calculations, which in turn influenced the corresponding cutoff values. As a result, our conclusions were drawn separately for each group, and any comparisons between groups were made qualitatively. Therefore, we maintain the perspective that the effect of uneven sample sizes on our results is relatively minimal.

## Data availability statement

The raw data supporting the conclusions of this article will be made available by the authors, without undue reservation.

## Ethics statement

The studies involving humans were approved by Hadassah Medical Center, Jerusalem, Israel, Ethics Committee and the Ethical Committee of the General University Hospital in Prague, Czech Republic. The studies were conducted in accordance with the local legislation and institutional requirements. The participants provided their written informed consent to participate in this study.

## Author contributions

GG: conception and design of the study, analysis developments, analysis, and writing of the manuscript. RD: young group data acquisition and preprocessing of all data. RJ: older group data acquisition and subject clinical evaluations. OB: older subject’s neuropsychological evaluations. All authors contributed to the article and approved the submitted version.

## Footnotes

^1^http://www.fil.ion.ucl.ac.uk/spm/software/spm12

^2^http://paos.colorado.edu/research/wavelets

## Abbreviations

BOLD: Blood oxygenation level-dependent
fMRI: functional MRI
