# Supplementary material for: Directed functional connectivity of the sensorimotor system in young and older individuals
Source: Front Aging Neurosci. 2023 Oct 11;15:1222352. doi: 10.3389/fnagi.2023.1222352 (PMC10597721; doi:10.3389/fnagi.2023.1222352)
Supplement: Supplementary file 1 [file Data_Sheet_1.docx]

Supplementary Information for:

**Directed Functional Connectivity of the Sensorimotor System in Young and Older Individuals**

**Table 1**. Neuropsychological battery used to assess cognitive function of Parkinson’s disease patients.

The neuropsychological battery consisted of ten tests in the following five domains, with one measure from each test derived as recommended for cognitive evaluation in Parkinson’s disease([Litvan et al., 2012](#_ENREF_5)). The following tests were used:

(1) Attention and working memory: Trail Making Test, part A([Bezdicek et al., 2012](#_ENREF_2)) and Digit span backwards from the Wechsler Adult Intelligence Scale, third revision (WAIS-III)([Wechsler, 1997](#_ENREF_12)).

(2) Executive function: Tower of London([Michalec, 2014](#_ENREF_6)) and semantic verbal fluency([Nikolai, 2015](#_ENREF_7)).

(3) Language: Boston Naming Test, Czech version([Tombaugh and Hubley, 1997](#_ENREF_11)) and WAIS-III Similarities([Wechsler, 1997](#_ENREF_12)).

(4) Memory: Rey Auditory Verbal Learning Test, delayed recall and Brief Visuospatial Memory Test, revised, delayed recall([Benedict et al., 1996](#_ENREF_1)).

(5) Visuospatial function: CLOX([Royall et al., 1998](#_ENREF_8)) and Judgment of Line Orientation([Woodard, 1998](#_ENREF_13))

**Table 2**. Results of the neuropsychological evaluation as a function of cognitive domain.

| Cognitive domain and tasks |  | Older subjects  (Mean ± SD) |
| --- | --- | --- |
| Psychomotor speed and working memory |  |  |
| Trail Making Test, part A (TMT-A) |  | 35.2±9.1 |
| Digit Span Backwards (DS back) |  | 6.7**±**2.2 |
| Executive function |  |  |
| Tower of London (ToL) |  | 25.9**±**3.8 |
| Semantic fluency: animals + clothes + shopping (COWAT) |  | 65.8**±**11.8 |
| Language |  |  |
| Boston Naming Test, Czech version (BNT-60) |  | 54.5**±**6.4 |
| Wechsler Adult Intelligence Scale, third revision, similarities (WAIS-III Sim) |  | 23.7**±**5.7 |
| Short term memory |  |  |
| Rey Auditory Verbal Learning Test, Delayed Recall (RAVLT-DR) |  | 8.9**±**2.4 |
| Brief Visuospatial Memory Test, Revised, Delayed Recall (BVMT-R-DR) |  | 10.2**±**1.7 |
| Visuospatial function |  |  |
| Royall’s CLOX (CLOX I) |  | 13.2**±**1.1 |
| Judgment of Line Orientation (JoL)  The raw scores (before transformation into a z-score) are provided for all tests. |  | 24.6**±**3.8 |

**Supplementary Table 3**.

Demographic and neuropsychological characteristics (z-scores and rankits) of older sample (N = 31)

| Demographics | Raw scores (M+SD (range)) | z-score (range) | Rankits (M+SD) |
| --- | --- | --- | --- |
| Age (years) | 63.2±7.89 (46-83) | - | - |
| Gender (male/female) | 15/16 | - | - |
| Education (years) | 14.8±3.5 (11-25) | - | - |
| MoCA | 26.6±2.2 (22-30) | -2.47-1.61 | -0.01±0.95 |
|  |  |  |  |
| Long-term memory |  |  |  |
| RAVLT-30 | 8.97±2.5 (2-12) | -1.99-1.21 | 0±0.98 |
| BVMT-R | 10.23±1.70 (5-12) | -2.91-.98 | -0.03±0.91 |
|  |  |  |  |
| Visuospatial function |  |  |  |
| Royall’s CLOX (CLOX I) | 13.26±1.15 (11-15) | -1.09-1.51 | -0.01±0.89 |
| Judgment of Line Orientation | 24.65±3.88 (11-29) | -1.45-1.11 | -0.01±0.95 |
|  |  |  |  |
| Psychomotor speed and working memory |  |  |  |
| Trail Making Test, part A | 35.2±9.1 (20-58) | -1.21-2.44 | 0±0.99 |
| WAIS-III Digit Span Backwards | 6.7±2.2 (4-12) | -1.17-2.20 | 0.02±0.93 |
|  |  |  |  |
| Executive function |  |  |  |
| Tower of London | 26.1±4.1 (16-34) | -2.51-2.04 | 0±0.99 |
| Semantic fluency | 65.6±11.8 (38-93) | -2.31-2.25 | 0±0.99 |
|  |  |  |  |
| Language |  |  |  |
| WAIS-III Similarities | 35.2±9.1 (20-58) | -2.16-1.41 | 0±0.99 |
| Boston Naming Test | 35.2±9.1 (20-58) | -1.91-.82 | -0.01±0.97 |

*Note*. Brief Visuospatial Learning Test—Revised; CLOX, Royall’s Clock Drawing Test; MoCA, Montreal Cognitive Assessment; RAVLT, Rey Auditory Verbal Learning Test; WAIS-III, Wechsler Adult Intelligence Scale—Third Revision.

**Supplementary Table 4**.

Pearson correlations of neuropsychological measures in the older sample (N = 31)

|  | | MoCA | Trails-A | Digit Span backwards | Tower of London | Semantic fluency | WAIS-III Similarities | BNT | RVLT delayed recall | BVMT delayed recall | Judgement of Line | CLOX-I |
| --- | --- | --- | --- | --- | --- | --- | --- | --- | --- | --- | --- | --- |
| MoCA | Pearson Correlation | 1 | .185 | .249 | -.122 | -.039 | .419^*^ | .239 | .306 | .554^**^ | .487^**^ | .358^*^ |
|  | Sig. (2-tailed) |  | .320 | .176 | .513 | .835 | .019 | .195 | .094 | .001 | .005 | .048 |
| Trails-A | Pearson Correlation | .185 | 1 | .019 | -.388^*^ | -.010 | .260 | .289 | .213 | -.103 | -.123 | .053 |
|  | Sig. (2-tailed) | .320 |  | .919 | .031 | .957 | .157 | .115 | .251 | .582 | .509 | .779 |
| Digit Span backwards | Pearson Correlation | .249 | .019 | 1 | .257 | .154 | .151 | .186 | .281 | .464^**^ | .210 | .154 |
|  | Sig. (2-tailed) | .176 | .919 |  | .163 | .409 | .417 | .316 | .125 | .009 | .256 | .408 |
| Tower of London | Pearson Correlation | -.122 | -.388^*^ | .257 | 1 | -.035 | -.086 | -.056 | .108 | .171 | -.296 | .084 |
|  | Sig. (2-tailed) | .513 | .031 | .163 |  | .851 | .646 | .765 | .563 | .358 | .106 | .652 |
| Semantic fluency | Pearson Correlation | -.039 | -.010 | .154 | -.035 | 1 | .159 | -.107 | .159 | .066 | -.214 | .061 |
|  | Sig. (2-tailed) | .835 | .957 | .409 | .851 |  | .393 | .567 | .392 | .724 | .249 | .746 |
| WAIS-III Similarities | Pearson Correlation | .419^*^ | .260 | .151 | -.086 | .159 | 1 | .173 | .373^*^ | .426^*^ | .223 | .480^**^ |
|  | Sig. (2-tailed) | .019 | .157 | .417 | .646 | .393 |  | .353 | .039 | .017 | .228 | .006 |
| BNT | Pearson Correlation | .239 | .289 | .186 | -.056 | -.107 | .173 | 1 | -.066 | -.014 | .121 | .212 |
|  | Sig. (2-tailed) | .195 | .115 | .316 | .765 | .567 | .353 |  | .726 | .940 | .517 | .253 |
| RVLT delayed recall | Pearson Correlation | .306 | .213 | .281 | .108 | .159 | .373^*^ | -.066 | 1 | .513^**^ | .033 | .119 |
|  | Sig. (2-tailed) | .094 | .251 | .125 | .563 | .392 | .039 | .726 |  | .003 | .860 | .525 |
| BVMT delayed recall | Pearson Correlation | .554^**^ | -.103 | .464^**^ | .171 | .066 | .426^*^ | -.014 | .513^**^ | 1 | .354 | .388^*^ |
|  | Sig. (2-tailed) | .001 | .582 | .009 | .358 | .724 | .017 | .940 | .003 |  | .051 | .031 |
| Judgement of Line | Pearson Correlation | .487^**^ | -.123 | .210 | -.296 | -.214 | .223 | .121 | .033 | .354 | 1 | .310 |
|  | Sig. (2-tailed) | .005 | .509 | .256 | .106 | .249 | .228 | .517 | .860 | .051 |  | .089 |
| CLOX-I | Pearson Correlation | .358^*^ | .053 | .154 | .084 | .061 | .480^**^ | .212 | .119 | .388^*^ | .310 | 1 |
|  | Sig. (2-tailed) | .048 | .779 | .408 | .652 | .746 | .006 | .253 | .525 | .031 | .089 |  |

**Table 5**

| **PW number** | **Pathway** |
| --- | --- |
| 1 | R1-R2-R3-X |
| 2 | R2-R3-X-R1 |
| 3 | R3-X-R1-R2 |
| 4 | X-R1-R2-R3 |
| 5 | R1-R2-X-R3 |
| 6 | R2-X-R3-R1 |
| 7 | R3-R1-R2-X |
| 8 | X-R3-R1-R2 |
| 9 | R1-R3-X-R2 |
| 10 | R3-X-R2-R1 |
| 11 | X-R2-R1-R3 |
| 12 | R2-R1-R3-X |
| 13 | R1-X-R3-R2 |
| 14 | R2-R1-X-R3 |
| 15 | R3-R2-R1-X |
| 16 | X-R3-R2-R1 |
| 17 | R1-R3-R2-X |
| 18 | R2-X-R1-R3 |
| 19 | R3-R2-X-R1 |
| 20 | X-R1-R3-R2 |
| 21 | R1-X-R2-R3 |
| 22 | R2-R3-R1-X |
| 23 | R3-R1-X-R2 |
| 24 | X-R2-R3-R1 |

**Table 5:** A list of continues pathways in a four-region pathway. R1 to R3 are three predefined seeds while X is a voxel.

| **Region 1** | **Region 2** | **Region 3** | **Region 4** |
| --- | --- | --- | --- |
| Precentral_L | Rolandic_Oper_L | Postcentral_L | Supp_Motor_Area_L |
| Precentral_L | Postcentral_L | Insula_R | Cingulum_Mid_L |
| Precentral_L | Postcentral_L | SupraMarginal_R | Cingulum_Mid_L |
| Precentral_L | SupraMarginal_R | Supp_Motor_Area_L | Calcarine_L |
| Precentral_L | Supp_Motor_Area_L | SupraMarginal_R | Cingulum_Mid_L |
| Precentral_L | Supp_Motor_Area_L | Postcentral_L | SupraMarginal_R |
| Precentral_L | Rolandic_Oper_L | Rolandic_Oper_R | Supp_Motor_Area_L |
| Precentral_L | Rolandic_Oper_R | SupraMarginal_R | Supp_Motor_Area_L |
| Cingulum_Mid_L | Postcentral_L | Supp_Motor_Area_L | Rolandic_Oper_R |
| Cingulum_Mid_L | Rolandic_Oper_L | Supp_Motor_Area_L | Postcentral_L |
| Cingulum_Mid_L | Insula_L | Lingual_R | Postcentral_L |
| Cingulum_Mid_L | Lingual_R | Calcarine_L | Precentral_L |
| Cingulum_Mid_L | Insula_L | Precentral_L | Supp_Motor_Area_L |
| Cingulum_Mid_L | Insula_R | Precentral_L | Supp_Motor_Area_L |
| Cingulum_Mid_L | Insula_L | Calcarine_L | Postcentral_L |
| Cingulum_Mid_L | Lingual_R | Calcarine_L | Rolandic_Oper_L |
| Insula_L | Insula_R | Precentral_L | SupraMarginal_R |
| Insula_L | Lingual_R | Rolandic_Oper_R | Precentral_L |
| Insula_L | Precentral_L | Rolandic_Oper_R | Supp_Motor_Area_L |
| Insula_L | Lingual_R | Calcarine_L | Precentral_L |
| Insula_L | Lingual_R | Rolandic_Oper_L | Postcentral_L |
| Insula_L | Lingual_R | Rolandic_Oper_R | Postcentral_L |
| Insula_R | Lingual_R | Calcarine_L | Precentral_L |
| Insula_R | Lingual_R | Rolandic_Oper_L | Precentral_L |
| Insula_R | Lingual_R | Rolandic_Oper_R | Precentral_L |
| Insula_R | Precentral_L | Rolandic_Oper_R | Supp_Motor_Area_L |
| Insula_R | Cingulum_Mid_L | Calcarine_L | Postcentral_L |
| Insula_R | Lingual_R | Precentral_L | Postcentral_L |
| Insula_R | Lingual_R | Rolandic_Oper_R | Postcentral_L |
| Insula_R | Rolandic_Oper_R | Cingulum_Mid_L | Postcentral_L |
| Insula_R | Rolandic_Oper_R | Postcentral_L | Supp_Motor_Area_L |
| Insula_R | Insula_L | Cingulum_Mid_L | Calcarine_L |
| Insula_R | Cingulum_Mid_L | Calcarine_L | Supp_Motor_Area_L |
| Insula_R | Insula_L | Calcarine_L | Supp_Motor_Area_L |
| Rolandic_Oper_L | Postcentral_L | SupraMarginal_R | Cingulum_Mid_L |
| Rolandic_Oper_R | Rolandic_Oper_L | Postcentral_L | SupraMarginal_R |
| Rolandic_Oper_L | Precentral_L | SupraMarginal_R | Cingulum_Mid_L |
| Rolandic_Oper_R | Postcentral_L | SupraMarginal_R | Cingulum_Mid_L |
| Supp_Motor_Area_L | Postcentral_L | SupraMarginal_R | Cingulum_Mid_L |
| Supp_Motor_Area_L | Postcentral_L | Rolandic_Oper_L | SupraMarginal_R |
| Calcarine_L | Precentral_L | Rolandic_Oper_R | Postcentral_L |
| Calcarine_L | Cingulum_Mid_L | Precentral_L | Rolandic_Oper_R |
| Calcarine_L | Cingulum_Mid_L | Precentral_L | Supp_Motor_Area_L |
| Calcarine_L | Lingual_R | Postcentral_L | SupraMarginal_R |
| Lingual_R | Rolandic_Oper_R | Precentral_L | Supp_Motor_Area_L |
| Lingual_R | Cingulum_Mid_L | Rolandic_Oper_R | Postcentral_L |
|  |  |  |  |

**Table 6:** List of pathways identified in the older subject group that correlated with apathy. The directionality is from left to right, starting from region 1, then progressing to region 2, region 3, and finally reaching region 4.

| **Region 1** | **Region 2** | **Region 3** | **Region 4** |
| --- | --- | --- | --- |
| Cingulum_Mid_L | Rolandic_Oper_R | Insula_L | Precentral_L |
| Cingulum_Mid_L | Precentral_L | Postcentral_L | Lingual_R |
| Cingulum_Mid_L | Precentral_L | Rolandic_Oper_R | Postcentral_L |
| Cingulum_Mid_L | Supp_Motor_Area_L | Precentral_L | Calcarine_L |
| Cingulum_Mid_L | Calcarine_L | Lingual_R | Insula_R |
| Rolandic_Oper_L | Precentral_L | Cingulum_Mid_L | SupraMarginal_R |
| Rolandic_Oper_R | Cingulum_Mid_L | Precentral_L | SupraMarginal_R |
| Rolandic_Oper_R | Supp_Motor_Area_L | Precentral_L | Postcentral_L |
| Rolandic_Oper_R | Rolandic_Oper_L | Supp_Motor_Area_L | Precentral_L |
| Rolandic_Oper_R | Cingulum_Mid_L | Postcentral_L | Lingual_R |
| Rolandic_Oper_R | Supp_Motor_Area_L | Rolandic_Oper_L | Postcentral_L |
| Supp_Motor_Area_L | Rolandic_Oper_R | Cingulum_Mid_L | Calcarine_L |
| Supp_Motor_Area_L | Cingulum_Mid_L | Postcentral_L | SupraMarginal_R |
| Supp_Motor_Area_L | Cingulum_Mid_L | Precentral_L | SupraMarginal_R |
| Insula_R | Insula_L | Postcentral_L | Cingulum_Mid_L |
| Insula_L | Rolandic_Oper_L | Postcentral_L | Cingulum_Mid_L |
| Insula_L | Rolandic_Oper_R | Rolandic_Oper_L | Postcentral_L |
| Insula_L | Calcarine_L | SupraMarginal_R | Insula_R |
| Lingual_R | SupraMarginal_R | Insula_L | Precentral_L |
| Lingual_R | SupraMarginal_R | Insula_R | Precentral_L |
| Lingual_R | SupraMarginal_R | Rolandic_Oper_L | Precentral_L |
| Lingual_R | Supp_Motor_Area_L | Insula_R | Postcentral_L |
| Lingual_R | SupraMarginal_R | Insula_R | Postcentral_L |
| Lingual_R | SupraMarginal_R | Rolandic_Oper_L | Postcentral_L |
| Lingual_R | Supp_Motor_Area_L | Rolandic_Oper_R | Postcentral_L |
| Lingual_R | Insula_L | Precentral_L | Postcentral_L |
| Lingual_R | SupraMarginal_R | Precentral_L | Postcentral_L |
| Precentral_L | Postcentral_L | Lingual_R | Calcarine_L |
| Precentral_L | Postcentral_L | Cingulum_Mid_L | SupraMarginal_R |
| Precentral_L | Rolandic_Oper_L | Cingulum_Mid_L | Calcarine_L |
| SupraMarginal_R | Cingulum_Mid_L | Precentral_L | Calcarine_L |

**Table 7:** List of pathways identified in the older subject group that correlated with Psychomotor speed and working memory. The directionality is from left to right, starting from region 1, then progressing to region 2, region 3, and finally reaching region 4
